# Polymorphism, Halogen Bonding, and Chalcogen Bonding in the Diiodine Adducts of 1,3- and 1,4-Dithiane

**DOI:** 10.3390/molecules26164985

**Published:** 2021-08-17

**Authors:** Andrew J. Peloquin, Srikar Alapati, Colin D. McMillen, Timothy W. Hanks, William T. Pennington

**Affiliations:** 1 Department of Chemistry, Clemson University, Clemson, SC 29634, USA; apeloqu@clemson.edu (A.J.P.); cmcmill@clemson.edu (C.D.M.); 2 Department of Chemistry, Furman University, Greenville, SC 29613, USA; srikar.alapati@furman.edu (S.A.); tim.hanks@furman.edu (T.W.H.)

**Keywords:** crystal engineering, chalcogen bonding, halogen bonding, polymorphism, X-ray diffraction

## Abstract

Through variations in reaction solvent and stoichiometry, a series of S-diiodine adducts of 1,3- and 1,4-dithiane were isolated by direct reaction of the dithianes with molecular diiodine in solution. In the case of 1,3-dithiane, variations in reaction solvent yielded both the equatorial and the axial isomers of S-diiodo-1,3-dithiane, and their solution thermodynamics were further studied via DFT. Additionally, S,S’-bis(diiodo)-1,3-dithiane was also isolated. The 1:1 cocrystal, (1,4-dithiane)·(I_2_) was further isolated, as well as a new polymorph of S,S’-bis(diiodo)-1,4-dithiane. Each structure showed significant S···I halogen and chalcogen bonding interactions. Further, the product of the diiodine-promoted oxidative addition of acetone to 1,4-dithiane, as well as two new cocrystals of 1,4-dithiane-1,4-dioxide involving hydronium, bromide, and tribromide ions, was isolated.

## 1. Introduction

Halogen bonding, and the related chalcogen bonding, interactions occur due to an interaction between the electrophilic region on a halogen or chalcogen atom within a molecule and a nucleophilic region on a different atom [1,2,3,4]. Due to their strength [5] and directionality [6], these interactions have emerged as useful tools in crystal engineering [7,8], pharmaceutical design [9,10,11], and in the understanding of synthetic transformations [12,13,14].

The reaction of diiodine, one of the simplest halogen bond donors, with thioethers allows the concomitant study of halogen and chalcogen bonding, as well as the competition between the two. Several notable examples of reaction products from diiodine with cyclic polythioethers exist in the literature, ranging from simple examples such as 1,4-dithaine [15] and 1,3,5-trithiane [16] to larger macrocycles [17,18,19]. A common structural motif that emerges is the presence of an S···I halogen bond of varying strength, with a combination of halogen and chalcogen bonding to consolidate packing in the crystalline state. The charge transfer component of these interactions has also been extensively characterized, with the interaction primarily attributed to an n→σ type interaction that increases in strength in the order of O < S < Se [20,21,22].

Herein, we report the isolation of a variety of crystalline polymorphs from the reaction of 1,3- and 1,4-dithiane with diiodine. In the case of 1,3-dithiane, two geometric isomers of S-diiodo-1,3-dithiane were isolated, with their solution behavior further studied via DFT computations, as well as S,S’-bis(diiodo)-1,3-dithiane. For 1,4-dithiane, the previously reported 1:1 cocrystal with I_2_ was isolated [15,23], as well as a new polymorph of S,S’-bis(diiodo)-1,4-dithiane. Each of these structures reveals an interplay between S···I halogen and chalcogen bonding. In the course of our investigations, we found that diiodine promoted oxidative addition of acetone to 1,4-dithiane, and we isolated two structures from the direct oxidation of the compound to dioxides, revealing alternative reaction pathways in these halogen-containing systems.

## 2. Results and Discussion

### 2.1. Reaction of 1,3-Dithiane with I_2_

**Figure 1:**
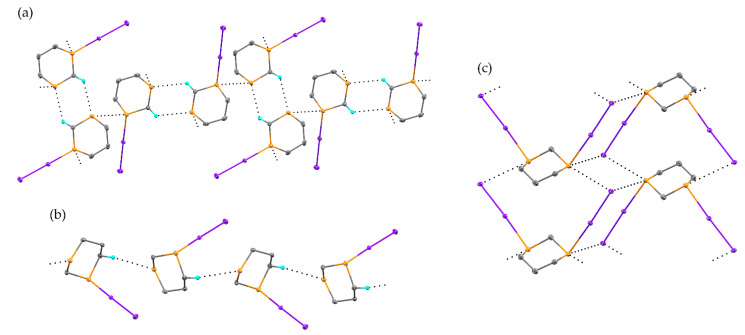
The solid-state structure of **1** (**a**), **2** (**b**), and **3** (**c**). Carbon atoms are gray. Sulfur atoms are yellow. Iodine atoms are purple. Hydrogen atoms are cyan. Intermolecular interactions are shown as black dotted lines. Only hydrogen atoms involved in the indicated intermolecular interactions are shown for clarity. Atomic displacement ellipsoids are shown at the 50% probability level.

**Figure 2:**
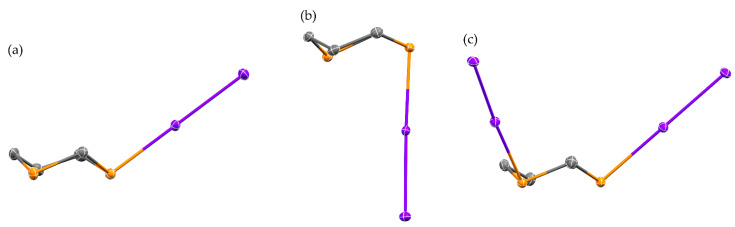
Single molecules of **1** (**a**), **2** (**b**), and **3** (**c**), arranged to show position of S-I-I relative to dithiane ring plane. Hydrogen atoms have been omitted for clarity. Atomic displacement ellipsoids are shown at the 50% probability level.

While the product of the reaction of 1,4-dithiane and I_2_ was first reported in the literature in 1960 [15,23], the analogous reaction of 1,3-dithiane has not been reported in the intervening 6 decades. Through variations in stoichiometry and solvent utilized for the reaction, we obtained three distinct products: S-diiodo-1,3-dithiane in both the equatorial (**1**) and axial (**2**) orientation of the diiodo pendant, as well as S,S’-bis(diiodo)-1,3-dithiane (**3**) (Figure 1 and Figure 2 and Figures S10–S12). When the reaction was conducted at a 1:1 stoichiometry in methanol, crystalline **1** was obtained, with the S-I-I group in a pseudo-equatorial position, with an angle of 52.29(3)° between I-I and the RMS ring plane (Table 1). The position of this substituent significantly deviates from the typical equatorial position (though not to the degree of the axial conformer **2** described below). For example, the analogous angle for an equatorial C-H in this molecule is only 8.43(16)°. Individual molecules pack in pairs in the solid-state via two C-H···S hydrogen bonds (*d*_H···S_ = 2.8840(17) Å, *R* = 0.93). These crystallographic dimers further interact to form sheets in the *bc* plane by an S···S contact involving an unsubstituted sulfur atom on one ring and the diiodo-substituted sulfur atom on a neighboring ring (*d*_S···S_ = 3.3571(6) Å, *R* = 0.89). Alternating dimers are rotated by approximately 72° to one another. The sheets stack in the *a* direction by a type I halogen interaction between terminal iodine atoms (*d*_I···I_ = 3.8019(3) Å, *θ*_C-H···S_ = 128.64(15)°).

When the 1:1 reaction of I_2_ and 1,3-dithiane was conducted in ethyl acetate, the axial isomer of S-diiodo-1,3-dithiane (**2**) was obtained. In this case, the S-I-I group is in a typical axial position, with an angle of 85.45(4)° between I-I and the RMS ring plane. The geometric change in the resulting molecule drastically affects the packing of the molecules in the solid-state. Chains are formed by weak C-H···S hydrogen bonds (*d*_H···S_ = 2.992(2) Å, *R* = 0.97), propagating in the *b* direction. Neighboring chains interact within the *ab* plane by S···S close contacts (*d*_S···S_ = 3.6110(8) Å, *R* = 0.96). The packing is further reinforced in the *c* direction by a bifurcated halogen bonding interaction, involving both a weak I···I (*d*_I···I_ = 3.9808(2) Å, *R* = 0.98) and I···S (*d*_I···S_ = 3.8928(5) Å, *R* = 0.99) contact. With the primary intermolecular interaction being of the weak C-H···S type, the decomposition temperature of **2**, 107 °C, is significantly lower than in **1**, 148 °C, where stronger S···S interactions are involved (Figures S1 and S2).

The product of the I_2_ addition to both sulfur atoms, S,S’-bis(diiodo)-1,3-dithiane (**3**), was obtained by reaction of 1,3-dithiane with two equivalents of I_2_ in methanol. In **3,** both S-I-I moieties are in pseudo-equatorial positions, with angles of 40.70(6)° and 49.07(7) between each I-I and the RMS ring plane. Double-stranded chains propagate in the *a* direction, formed by a cooperative series of S···I chalcogen bonds (*d*_S···I_ = 3.8445(8) Å and 3.8712(8) Å, *R* = 0.98 and 0.99).

### 2.2. Computational Investigation of S-Diiodo-1,3-Dithiane Ring Flip

**Figure 3:**
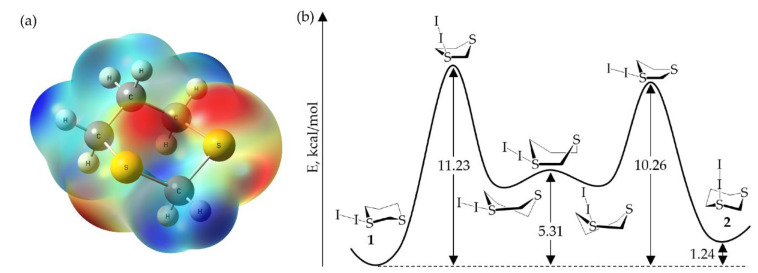
Electrostatic potential (ESP) map of 1,3-dithiane (**a**) and reaction coordinate of the ring flip from **1** to **2** (**b**). The electrostatic potential is calculated at the 0.005 au isodensity surface and ranges from −25 kcal/mol (red) to 38 kcal/mol (blue).

The electrostatic potential map of 1,3-dithiane, generated by DFT calculations, shows distinct regions of negative electrostatic potential around sulfur, as would be expected from a sp^3^ hybridized atom. These are localized both above and below the ring plane (Figure 3a). Either region could potentially serve as a hydrogen or halogen bond acceptor. This is seen in structures **1**–**3**, with **1** having a pseudo equatorial halogen bond to diiodine, while **2** and **3** have axial halogen bonds.

The conformations and inversion pathways of 1,3- and 1,4-dithiane have previously been examined by gas-phase DFT calculations [24,25,26]. In general, the energetics of these processes were found to be similar to those of cyclohexane, which has been thoroughly studied both experimentally and computationally. The inversion of the 1,3-dithiane ring was reported to proceed through twisted boat intermediates, lying 4.72 kcal/mol above the chair confirmation (slightly lower than the equivalent cyclohexane intermediates). The calculated transition states for this process were found to be 9.87 kcal/mol above the chair state, while the boat transition state between the two intermediate structures was only 0.80 kcal/mol higher in energy. The interconversion of the equatorial and axial isomers of 1,3-dithiane/I_2_ can proceed through multiple pathways since the addition of I_2_ at only one sulfur breaks the symmetry of the system. Gas-phase calculations of one of the inversion pathways of the S-diiodo substituted system indicate a ΔE‡ of 11.23 kcal/mol between **1** and its respective half-chair conformer. Conformer **2** lies only slightly higher in energy than **1** (ΔE = 1.24 kcal/mol), with a ΔE‡ of 9.02 kcal/mol between **2** and its respective half chair. The S-I binding energy remains relatively consistent throughout the ring inversion, at approximately 11 kcal/mol. The calculated energy differences remain consistent relative to the gas-phase when solvation in methanol or ethyl acetate was included via SMD. These results indicate the preferential formation of **1** or **2** is not driven by conformational equilibrium in solution.

### 2.3. Reaction of 1,4-Dithiane with I_2_

**Figure 4:**
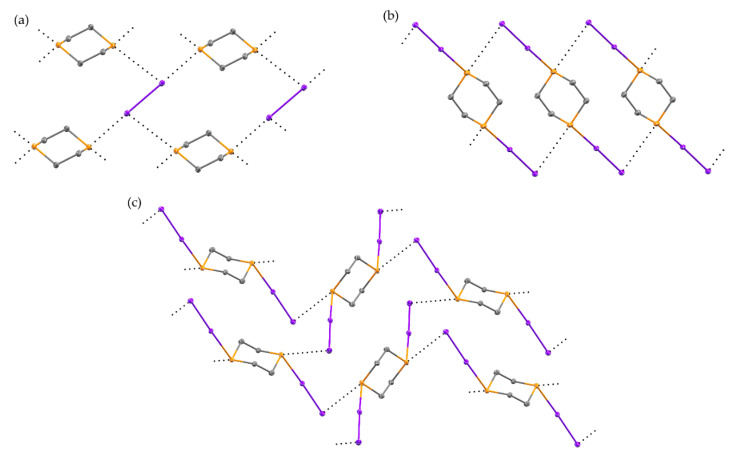
The solid-state structure of **4** (**a**), **5** (**b**), and **6** (**c**). Intermolecular interactions are shown as black dotted lines. Hydrogen atoms have been omitted for clarity. Atomic displacement ellipsoids are shown at the 50% probability level.

Through variations in solvent and stoichiometry, three new structures resulting from the reaction of I_2_ and 1,4-dithiane were obtained. The first, (1,4-dithiane)(I_2_) (**4**), is a 1:1 cocrystal obtained when the reaction is conducted in perfluoropyridine, and it possesses a significantly longer S···I distance than any of the other products observed in this study (Figure 4a and Supplementary Figure S13), such that the S···I interactions in **4** distinguish themselves from the more covalently bonded adducts of the other structures (**1**–**3**, **5**, **6**). Each iodine atom is involved in both a strong halogen bond (*d*_S···I_ = 3.0813(7) Å, *R* = 0.78) and much weaker chalcogen bond (*d*_S···I_ = 3.8728(7) Å, *R* = 0.99). The two types of interactions can be easily distinguished by the orientation of the interaction relative to the I-I bond. For the halogen bond, the S···I-I angle of 179.074(12)° indicates involvement of the σ hole on the iodine atom [27,28,29]. The chalcogen bond shows a distinctly different orientation, with an S···I-I angle of 77.077(14)°, as well as a C-S···I angle of 177.17(8)°. These two angles taken together demonstrate an interaction between the band of increased electrostatic potential on the iodine atom, located normal to the I-I bond, as well as the σ hole of the sulfur atom, located along the elongation of the C-S bond.

While increasing the iodine available for reaction to 2:1 diiodine:dithiane, variations in reaction solvent again produced polymorphic structures with 1,4-dithiane (Figure 4b,c and Figures S14 and S15) however, in this case, the polymorphic structures are of S,S’-bis(diiodo)-1,4-dithiane. When the reaction was conducted in methanol, structure **5** formed. This is isostructural to the IBr adduct, with an elongation of the S-I bond from 2.687(2) Å in the IBr compound to 2.7891(6) Å in **5** [30]. The packing is dominated by S···I chalcogen bonds (*d*_S···I_ = 3.7591(6) Å, *R* = 0.96, *θ*_C–S···I_ = 176.81(9)°), which contribute to the formation of ribbons in the *ab* plane. Neighboring ribbons interact through a weak type I interaction between terminal iodine atoms. To provide the most accurate comparison to the McCollough (1,4-dithiane)(I_2_)_2_ polymorph, the reaction was also conducted in dichloromethane, yielding polymorph **6**, with the same reduced unit cell parameters as those previously reported [15]. While the overall packing arrangement remains consistent, the reduction in data collection temperature causes a noticeable reduction in intermolecular contact distances. The S···I distance is 2.8036(7) in **6**, reduced from 2.87 Å in the room temperature structure. A network of chalcogen bonds (*d*_S···I_ = 3.6692(7) Å, *R* = 0.93, *θ*_C-S···I_ = 166.55(9)°) promotes the formation of sheets within the (1 0 1) plane. This distance is again reduced from the room temperature structure (3.78 Å). Neighboring sheets stack by weak S···I halogen bonds (*d*_S···I_ = 3.9907(7) Å, *R* = 1.02). The arrangement of S-I-I moiety is pseudo in both **5** and **6**, with an angle from the dithiane RMS ring plane of 47.22(5)° and 42.60(5)° respectively. Surprisingly, despite significant differences in the primary halogen bonding interactions between **4**, **5**, and **6**, the decomposition temperatures are within 7 °C of one another (Figures S4–S6).

### 2.4. Other Dihalogen Promoted Reactions of 1,4-Dithiane

**Figure 5:**
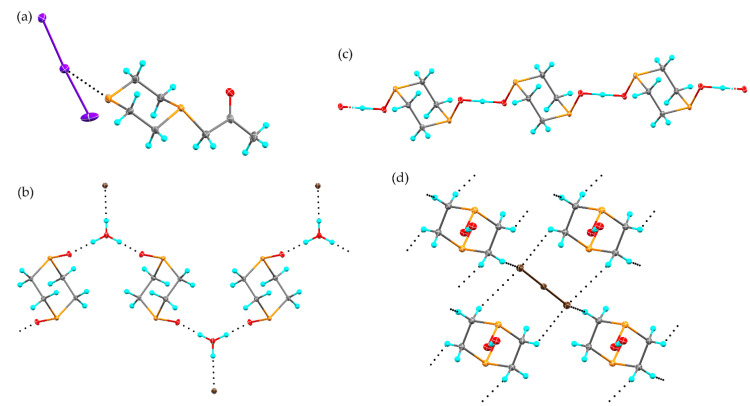
The solid-state structure of **7** (**a**), **8** (**b**), and **9** (**c**,**d**). Both the O···H···O hydrogen-bonded chains (**c**) and connection of neighboring chains by C-H···Br hydrogen bonding (**d**) are shown. Bromine atoms are brown. Oxygen atoms are red. Intermolecular interactions are shown as black dotted lines. Atomic displacement ellipsoids are shown at the 50% probability level.

Throughout this investigation, several other interesting products that involve the formation of new covalent bonds to sulfur were obtained by the reaction of 1,4-dithiane with both I_2_ and Br_2_. If the 2:1 I_2_:1,4-dithiane reaction is conducted in acetone, a sulfonium cation is formed from the addition of acetone to one of the sulfur atoms of the dithiane ring (**7**) (Figure 5a and Figure S16). A triiodide anion is also present to balance the charge. A chalcogen bond is present between the disubstituted sulfur atom and the central atom of the triiodide anion (*d*_S···I_ = 3.8586(7) Å, *R* = 0.98, *θ*_C-S···I_ = 177.55(10)°). The bromide salt of this compound has been prepared previously by the reaction of bromoacetone and 1,4-dithiane in ethanol [31].

First reported in 1927, the reaction of 1,4-dithiane with Br_2_ provides 1,4-dithiane-S,S’-dioxide [32]. The crystalline structure of this molecule in isolation has been further reported twice in the literature [33,34]. While our attempts to investigate the halogen bonding of Br_2_ with 1,4-dithiane were unsuccessful, resulting in the dioxide due to the increased oxidizing power of Br_2_ relative to I_2_, two new structures containing 1,4-dithiane-S,S’-dioxide were obtained. The first, (O=S(CH_2_CH_2_)_2_S=O)(H_3_O)(Br) (**8**), was obtained by the reaction of 1,4-dithiane with Br_2_ in a 5:1 mixture of methanol:acetone (Figure 5b). Acetone is required to resolubilize the dioxide which immediately precipitates from the solution upon Br_2_ addition. The resulting crystalline material showcases chains formed by hydrogen bonding between oxygen atoms on two separate 1,4-dithiane-S,S’-dioxide molecules and two hydrogen atoms of a hydronium ion. The third hydronium hydrogen atom participates in a hydrogen bond to a bromide anion. In contrast to **8**, the reaction of 1,4-dithiane with Br_2_ in acetonitrile produced crystalline (O=S(CH_2_CH_2_)_2_S=O-H)(Br_3_) (**9**). Resulting from the single protonation of a S=O oxygen atom, likely from HBr formed from water contamination of Br_2_, the mixed sulfoxide/sulfoxonium cations form 1-D chains along the *b* axis (Figure 5c and Figure S18). The single −OH hydrogen atom is equally shared between two oxygen atoms. Four neighboring chains are consolidated through a series of C-H···Br hydrogen bonds to the tribromide anion.

## 3. Materials and Methods

### 3.1. Materials

1,3-dithiane (>97%, CAS registry no. 505-23-7) was obtained from Oakwood Chemical (Estill, SC, USA). 1,4-dithiane (>97%, CAS registry no. 505-29-3) was obtained from Millipore Sigma (St. Louis, MO, USA). Iodine (>99.8%, resublimed, CAS registry number 7553-56-2), bromine (>99.8%, CAS registry no. 7726-95-6), methanol (100%, CAS registry no. 64-56-1), and ethyl acetate (>99.5%, CAS registry no. 141-78-6) were obtained from Fisher Scientific (Waltham, MA, USA). Pentafluoropyridine (99%, CAS registry no. 700-16-3) was obtained from Synquest Laboratoires (Alachua, FL, USA). All materials were used as received.

### 3.2. Thermal Analysis

Simultaneous thermal gravimetric analysis (TGA) and differential scanning calorimetry (DSC) measurements were made using a TA Instruments Q600 or Discovery 650 (New Castle, DE, USA). Samples were heated from room temperature to 500 °C in air (100 mL/min) at a rate of 10 °C/min.

### 3.3. Single-Crystal X-ray Analysis

Single crystal X-ray diffraction data were collected using a Bruker D8 Venture diffractometer (Madison, WI, USA) equipped with Mo Kα radiation (λ = 0.71073 Å) and a Photon 100 detector. Data were collected in 0.5° oscillations of phi and/or omega at a temperature of 100 K. Data were processed and corrected for absorption using the SAINT and SADABS routines in the Apex 3 software suite (v2017.3, Madison, WI, USA, 2017) [35]. The structures were solved by intrinsic phasing (SHELXT) [36] and subsequently refined within Olex2 [37,38]. Non-hydrogen atoms were refined anisotropically, and hydrogen atoms attached to carbon atoms were refined in calculated positions using riding models. Hydrogen atoms attached to oxygen atoms in **8** and **9** were located in the electron difference map and freely refined. Appropriate DFIX constraints were used in **9**. Crystallographic data are reported in Table S1.

### 3.4. Computational Study of ***1*** and ***2***

All DFT calculations were conducted using the Gaussian 09 package (B.01, Wallingford, CT, USA, 2010) [39]. Structures **1**, **2**, and their respective twisted boat conformations were optimized using the M06-2X functional and cc-pVTZ basis set with the LANL2DZ pseudopotential [40,41,42,43]. These optimized structures were used as starting points in the qst3 method to find the transition states. Frequency calculations were performed using M06-2X/cc-pVTZ with LANL2DZ pseudopotential at 298.15 K and 1 atm of pressure. Each optimized transition state structure had one imaginary frequency and was used in intrinsic reaction coordinate (IRC) calculations to examine the reaction path of the transition state.

### 3.5. Synthesis of Cocrystals

The synthesis of all cocrystals was scaled for a maximum yield of 100 to 200 mg of the desired product. Reagents were dissolved at the indicated ratios in a minimum amount of solvent and allowed to evaporate slowly at room temperature. Vials were sealed to halt evaporation as soon as crystals were observed to ensure sample purity.

*S-diiodo-1,3-dithiane (equatorial)* (**1**). 1,3-dithiane (44 mg, 0.37 mmol) and diiodine (93 mg, 0.37 mmol) were dissolved in methanol (10 mL) and the solvent was allowed to slowly evaporate at room temperature. T_decomp_ 148 °C. Anal Calcd for C_4_H_8_I_2_S_2_ (374.0): C, 12.84; H, 2.16; S, 17.15; Found: C, 12.65; H, 2.13; S, 16.88.

*S-diiodo-1,3-dithiane (axial)* (**2**). 1,3-dithiane (37 mg, 0.31 mmol) and diiodine (78 mg, 0.31 mmol) were dissolved in ethyl acetate (10 mL) and the solvent was allowed to slowly evaporate at room temperature. T_decomp_ 107 °C. Anal Calcd for C_4_H_8_I_2_S_2_ (374.0): C, 12.84; H, 2.16; S, 17.15; Found: C, 13.10; H, 2.36; S, 17.12.

*S,S′-bis(diiodo)-1,3-dithiane* (**3**). 1,3-dithiane (26 mg, 0.22 mmol) and diiodine (110 mg, 0.44 mmol) were dissolved in methanol (10 mL) and the solvent was allowed to slowly evaporate at room temperature. T_decomp_ 115 °C. Anal Calcd for C_4_H_8_I_4_S_2_ (627.9): C, 7.65; H, 1.28; S, 10.21; Found: C, 7.80; H, 1.04; S, 10.40.

*1,4-dithiane·I_2_* (**4**). 1,4-dithiane (34 mg, 0.28 mmol) and diiodine (72 mg, 0.28 mmol) were dissolved in perfluoropyridine (10 mL) and the solvent was allowed to slowly evaporate at room temperature. T_decomp_ 126 °C. Anal Calcd for C_4_H_8_I_2_S_2_ (374.0): C, 12.84; H, 2.16; S, 17.15; Found: C, 12.65; H, 2.46; S, 16.81.

*S,S′-bis(diiodo)-1,4-dithiane* (**5**). 1,4-dithiane (28 mg, 0.23 mmol) and diiodine (118 mg, 0.27 mmol) were dissolved in methanol (10 mL) and the solvent was allowed to slowly evaporate at room temperature. T_decomp_ 129 °C. Anal Calcd for C_4_H_8_I_4_S_2_ (627.9): C, 7.65; H, 1.28; S, 10.21; Found: C, 7.71; H, 1.14; S, 10.46.

*S,S′-bis(diiodo)-1,4-dithiane* (**6**). 1,4-dithiane (31 mg, 0.26 mmol) and diiodine (131 mg, 0.52 mmol) were dissolved in methanol (10 mL) and the solvent was allowed to slowly evaporate at room temperature. T_melt_ 62 °C. T_decomp_ 122 °C. Anal Calcd for C_4_H_8_I_4_S_2_ (627.9): C, 7.65; H, 1.28; S, 10.21; Found: C, 7.77; H, 1.29; S, 10.45.

*1-(2-oxopropyl)-1,4-dithianium triiodide* (**7**). 1,4-dithiane (34 mg, 0.28 mmol) and diiodine (144 mg, 0.57 mmol) were dissolved in acetone (10 mL) and the solvent was allowed to slowly evaporate at room temperature. T_decomp_ 126 °C. Anal Calcd for C_7_H_13_I_3_OS_2_ (558.0): C, 15.07; H, 2.35; S, 11.49; Found: C, 14.72; H, 2.05; S, 11.34.

*1,4-dithiane-S,S′-dioxide hydronium bromide* (**8**). 1,4-dithiane (112 mg, 0.93 mmol) was dissolved in methanol (10 mL) and bromine was added (0.25 mL, 4.6 mmol), resulting in immediate precipitation of a white solid. Acetone (5 mL) was added to resolubilize the precipitate and the resulting solvent mixture was allowed to slowly evaporate at room temperature. T_decomp_ 96 °C. Anal Calcd for C_4_H_11_BrO_3_S_2_ (251.2): C, 19.13; H, 4.41; S, 25.53; Found: C, 19.26; H, 4.40; S, 25.92.

*1,4-dithiane-S-oxide-S′-oxonium tribromide* (**9**). 1,4-dithiane (141 mg, 0. 17 mmol) was dissolved in acetonitrile (10 mL) and bromine was added (0.31 mL, 5.9 mmol). The solvent was then allowed to slowly evaporate at room temperature. T_decomp_ 98 °C. Anal Calcd for C_4_H_9_Br_3_O_2_S_2_ (393.0): C, 12.23; H, 2.31; S, 16.32; Found: C, 11.99; H, 1.98; S, 16.37.

## Figures and Tables

**Table 1:** S···I-I interaction distances (Å) and angles (°).

Crystal	*d*_S-I_	*d*_I-I_	*θ*_S-I-I_	*θ*_ring-I-I_^a^
**1**	2.7124(4)	2.85193(18)	179.438(9)	52.29(3)
**2**	2.7465(6)	2.8364(2)	176.579(12)	85.45(4)
**3**	2.8135(9)	2.8072(4)	177.140(18)	40.70(6)
	2.8329(9)	2.7874(4)	176.229(18)	49.07(7)
**4**	3.0813(7)	2.7827(4)	179.074(12)	38.25(5)
**5**	2.7891(6)	2.8117(3)	178.576(11)	42.78(5)
**6**	2.8036(7)	2.8031(3)	177.426(16)	47.40(5)

a: angle calculated between I-I bond and RMS plane of dithiane ring.

## Data Availability

Crystal data for **1**–**9** were deposited with the Cambridge Crystallographic Data Center (CCDC) (https://www.ccdc.cam.ac.uk, accessed on 17 august 2021) with deposition numbers 2097610–2097618.

## Supplementary Materials

The following are available online. Figures S1–S9, Simultaneous differential scanning calorimetry and thermal gravimetric analysis (SDT) of **1**–**9**, Figures S10–S18, Unit cell packing diagrams of **1**–**9**, Table S1, Crystallographic data and selected data collection parameters.

## Sample Availability

Samples of the compoundsare not available from the authors.
