# Supplementary material for: Polymorphism, Halogen Bonding, and Chalcogen Bonding in the Diiodine Adducts of 1,3- and 1,4-Dithiane
Source: Molecules. 2021 Aug 17;26(16):4985. doi: 10.3390/molecules26164985 (PMC8398952; doi:10.3390/molecules26164985)
Supplement: Supplementary file 1 [file molecules-26-04985-s001.zip › molecules-1335613-supplementary.pdf]

Supporting information

Polymorphism and Halogen Bonding in the diiodine adducts of 1,3- and 1,4-dithiane

Andrew J. Peloquin[1], Srikar Alapati[2], Colin D. McMillian[1], Timothy W. Hanks[2] and William T. Pennington[1,*]

[1]Department of Chemistry, Clemson University, Clemson, SC

[2]Department of Chemistry, Furman University, Greenville, SC

Table of Contents

Figure S1. SDT of **1** SI2
Figure S2. SDT of **2** SI2
Figure S3. SDT of **3** SI3
Figure S4. SDT of **4** SI3
Figure S5. SDT of **5** SI4
Figure S6. SDT of **6** SI4
Figure S7. SDT of **7** SI5
Figure S8. SDT of **8** SI5
Figure S9. SDT of **9** SI6

Table S1. Crystallograhic data and data collection parameters........ SI7–8

Figure S10. Unit cell packing of **1** SI9
Figure S11. Unit cell packing of **2** SI9
Figure S12. Unit cell packing of **3** SI10
Figure S13. Unit cell packing of **4** SI10
Figure S14. Unit cell packing of **5** SI11
Figure S15. Unit cell packing of **6** SI11
Figure S16. Unit cell packing of **7** SI12
Figure S17. Unit cell packing of **8** SI12
Figure S18. Unit cell packing of **9** SI13

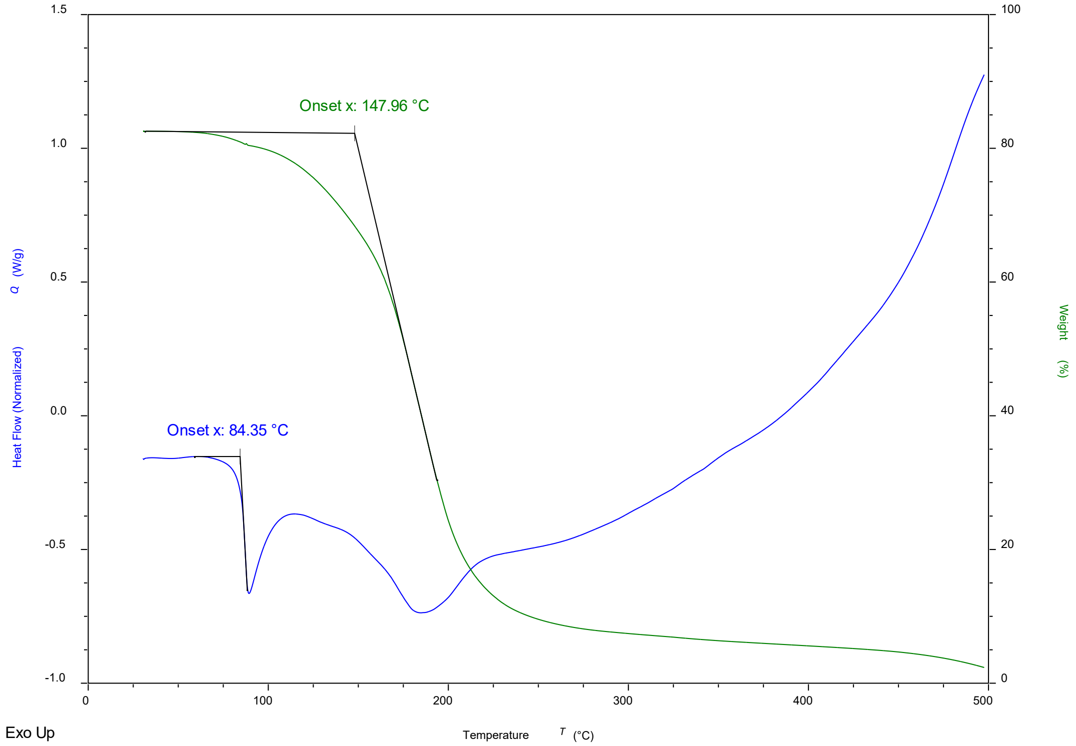

Figure S1. Simultaneous differential scanning calorimetry (blue) and thermal gravimetric analysis (green) of **1**

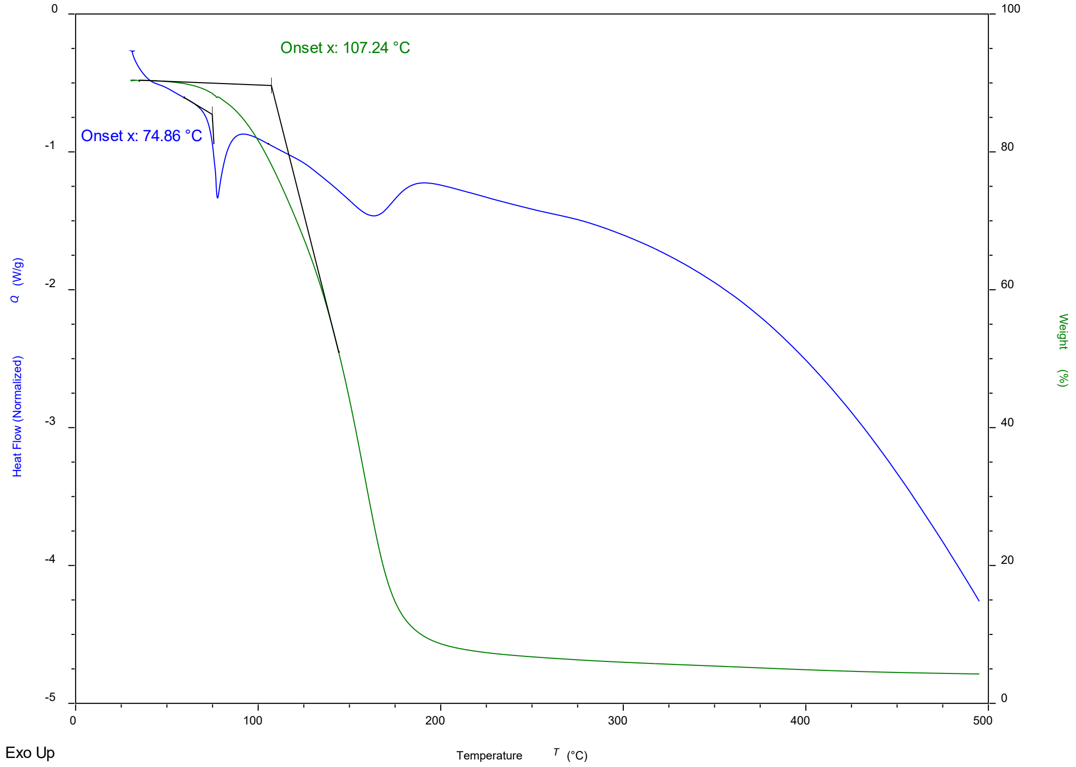

Figure S2. Simultaneous differential scanning calorimetry (blue) and thermal gravimetric analysis (green) of **2**

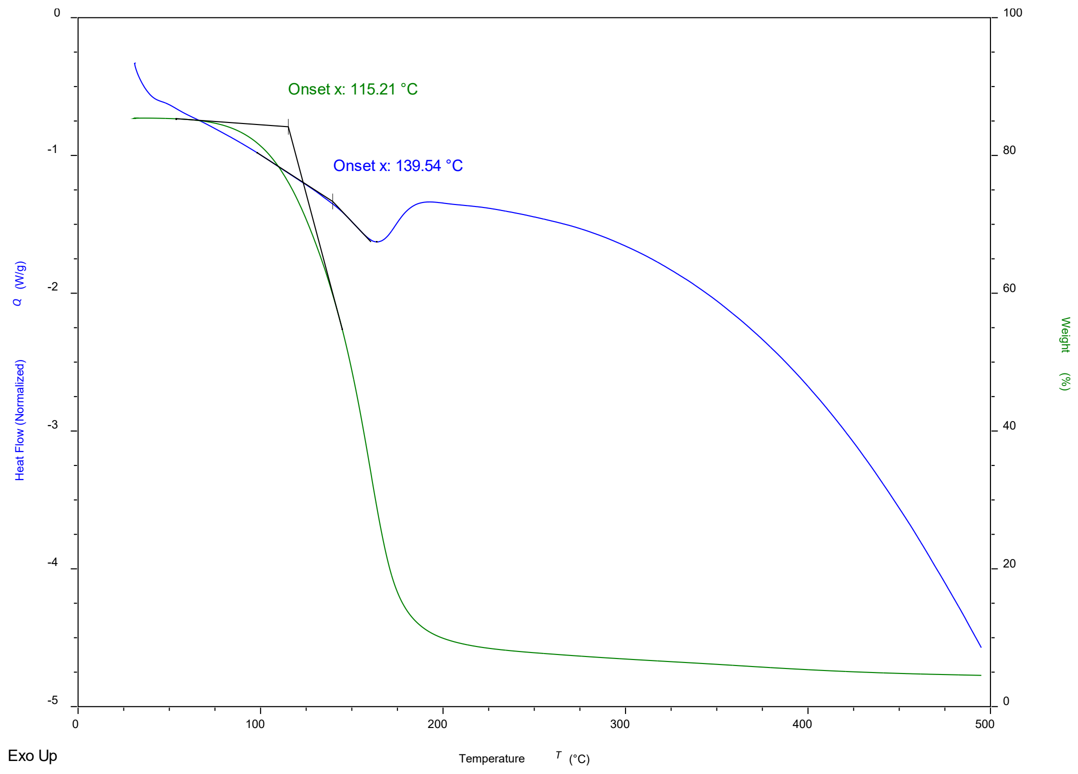

Figure S3. Simultaneous differential scanning calorimetry (blue) and thermal gravimetric analysis (green) of **3**

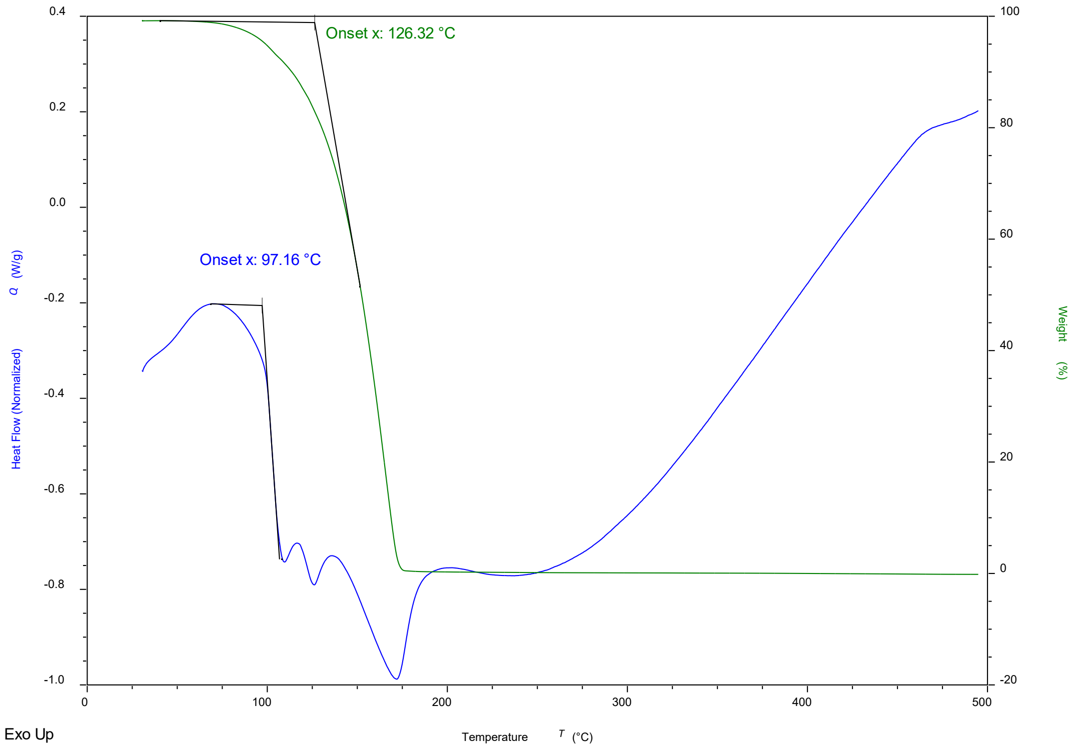

Figure S4. Simultaneous differential scanning calorimetry (blue) and thermal gravimetric analysis (green) of **4**

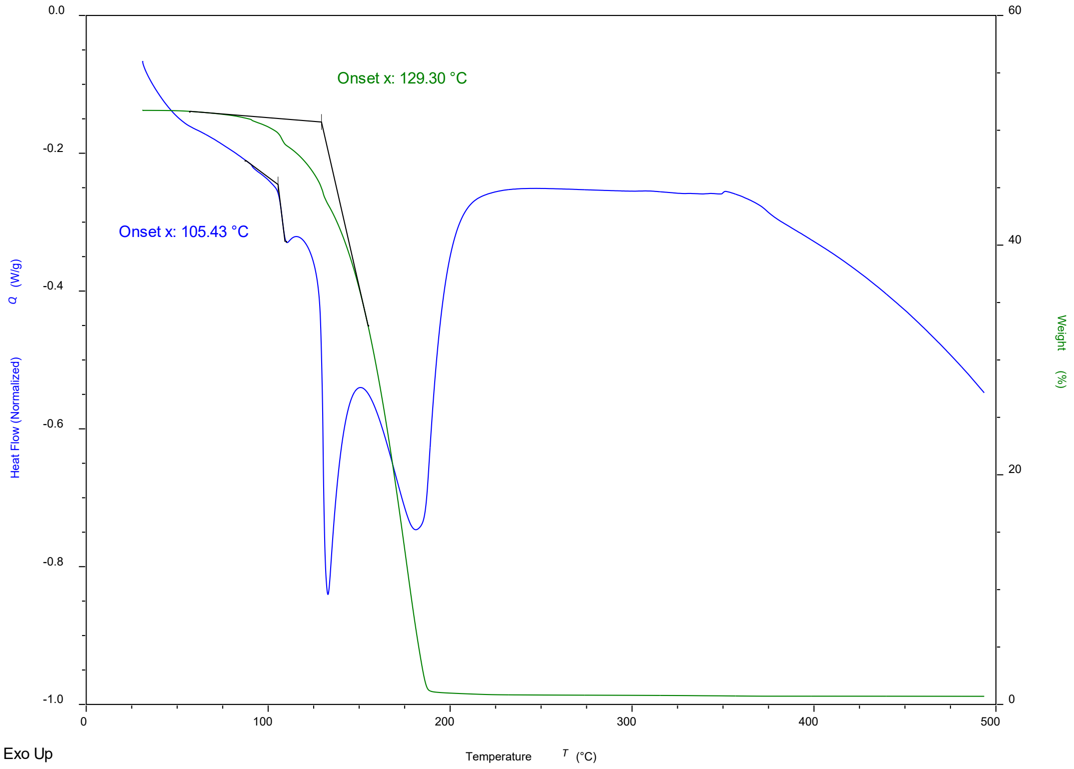

Figure S5. Simultaneous differential scanning calorimetry (blue) and thermal gravimetric analysis (green) of **5**

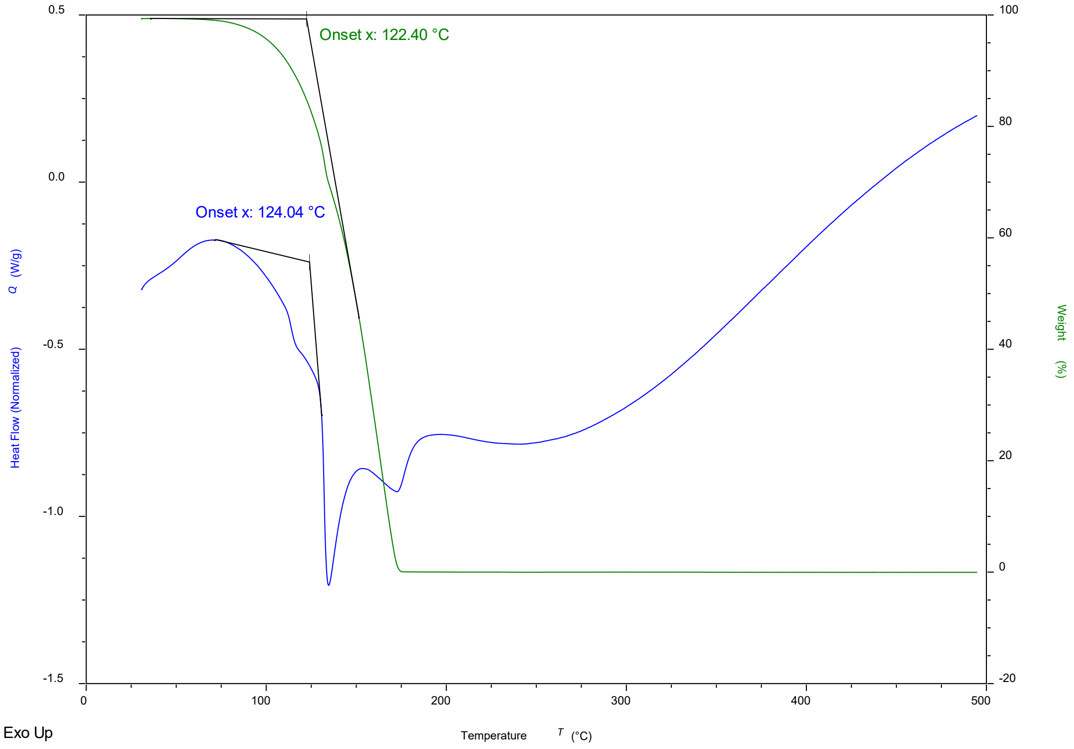

Figure S6. Simultaneous differential scanning calorimetry (blue) and thermal gravimetric analysis (green) of **6**

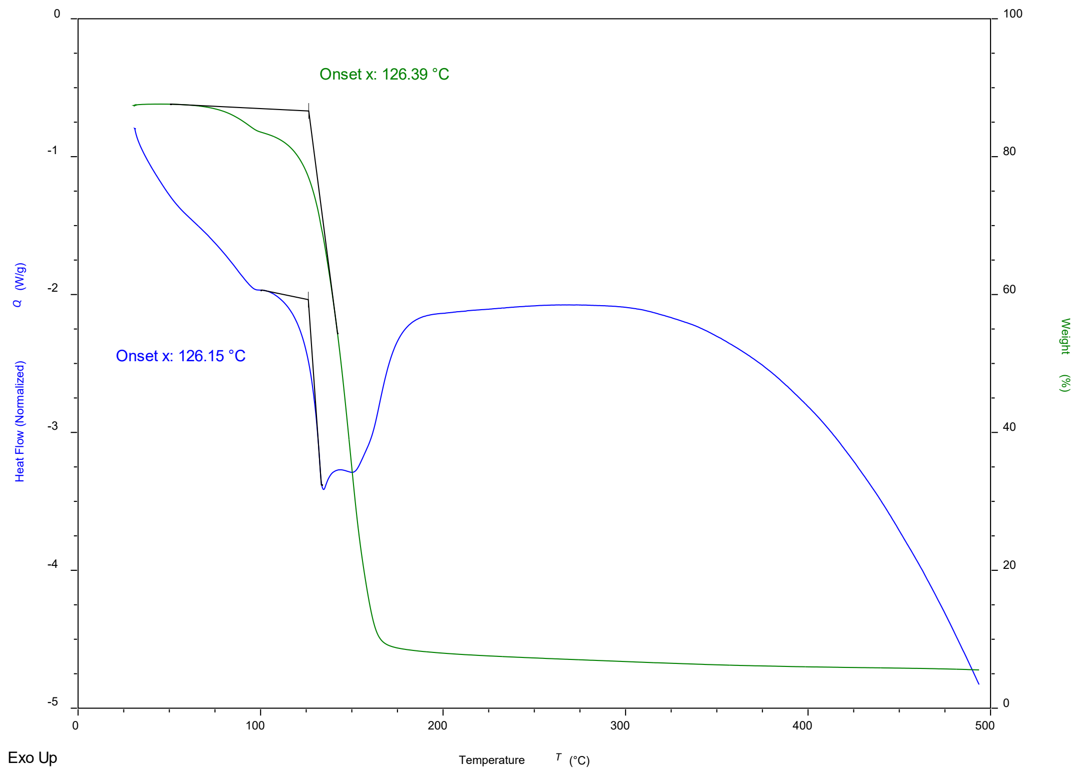

Figure S7. Simultaneous differential scanning calorimetry (blue) and thermal gravimetric analysis (green) of **7**

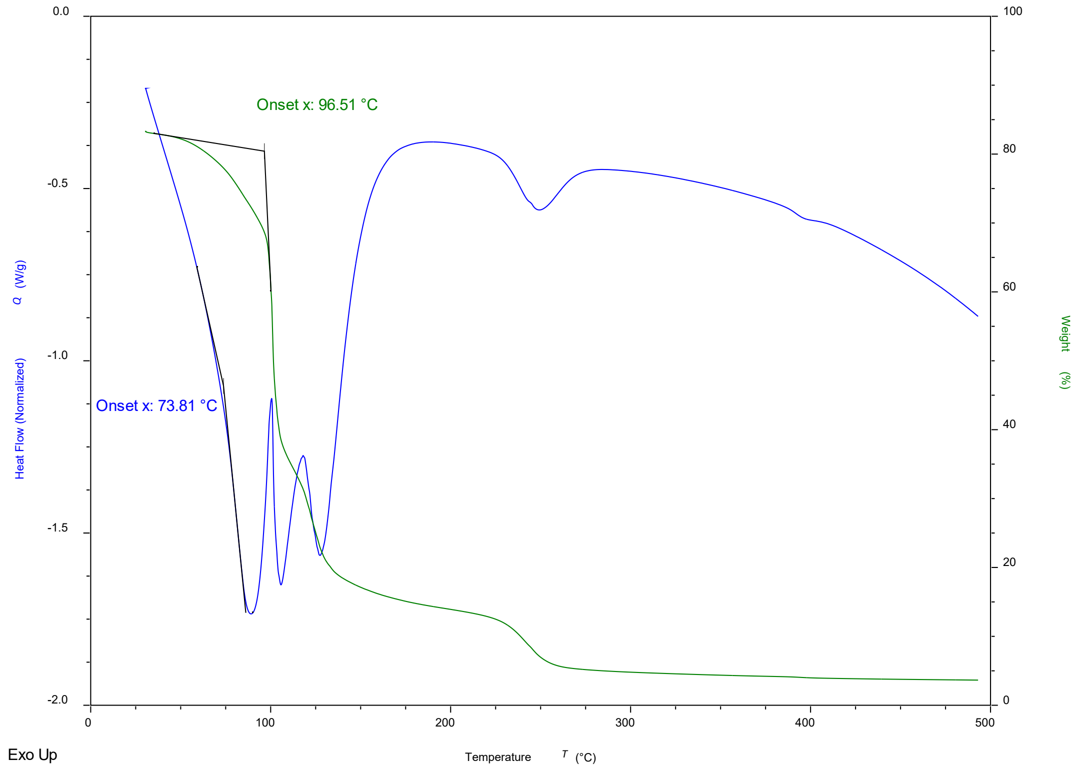

Figure S8. Simultaneous differential scanning calorimetry (blue) and thermal gravimetric analysis (green) of **8**

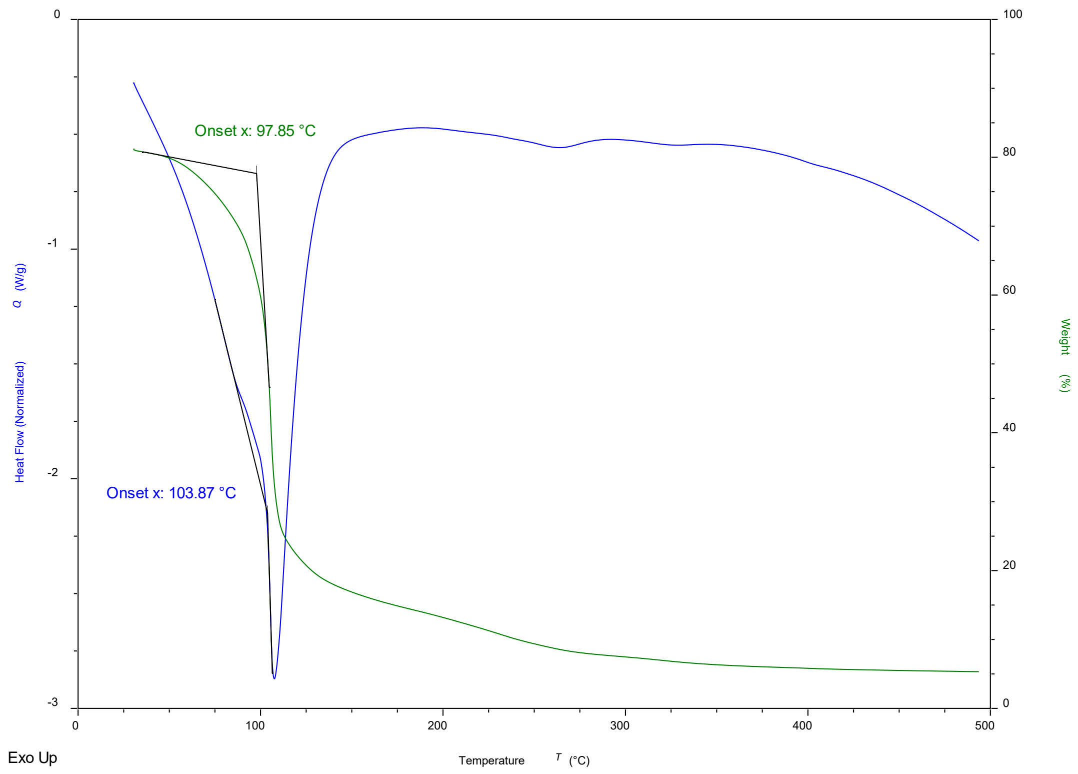

Figure S9. Simultaneous differential scanning calorimetry (blue) and thermal gravimetric analysis (green) of **9**

Table S1. Crystallographic data and selected data collection parameters

Cocrystal	1,3-dithiane-S-I_2 (equatorial) (1)	1,3-dithiane-S-I_2 (axial) (2)	1,3-dithiane-2(S-I_2) (3)	1,4-dithiane-S-I_2 (4)	1,4-dithiane-2(S-I_2) polymorph #1 (5)	1,4-dithiane-2(S-I_2) polymorph #2 (6)
empirical formula	$C_4H_8I_2S_2$	$C_4H_8I_2S_2$	$C_4H_8I_4S_2$	$C_4H_8I_2S_2$	$C_4H_8I_4S_2$	$C_4H_8I_4S_2$
crystal system	monoclinic	orthorhombic	monoclinic	triclinic	monoclinic	monoclinic
space group	$P2_1/c$	*Pbca*	$P2_1/c$	*P*-1	$P2_1/c$	$P2_1/n$
a (Å)	8.0519(5)	11.0715(3)	6.0638(4)	5.6537(8)	6.1527(5)	6.6951(4)
b (Å)	8.8801(5)	11.8356(3)	24.0060(16)	6.2843(9)	11.9103(10)	6.3650(3)
c (Å)	13.0503(7)	13.7461(3)	8.7795(6)	6.8239(9)	8.7326(8)	14.9858(8)
α (°)	90	90	90	100.032(5)	90	90
β (°)	97.043(2)	90	99.174(2)	94.296(6)	101.442(3)	90.318(2)
γ (°)	90	90	90	94.952(6)	90	90
Volume (Å^3)	926.08(9)	1801.26(8)	1261.66(15)	236.85(6)	627.21(9)	638.60(6)
Z	4	8	4	1	2	2
D_c (g·cm^{-3})	2.683	2.759	3.305	2.622	3.325	3.265
μ (mm^{-1})	7.159	7.361	10.156	6.998	10.215	10.032
No. of ref. total	27748	10088	25857	6073	9824	9163
No. of ref. unique	1785	1828	2578	1090	1267	1275
No. of param.	73	73	91	37	46	46
R_1	0.0096	0.0131	0.0158	0.0166	0.0112	0.0136
$R_{1\mathrm{all}}$	0.0098	0.0146	0.0174	0.0168	0.0262	0.0138
$_{\mathrm{w}}R_2$	0.0222	0.0266	0.0372	0.0417	0.0114	0.0309
$_{\mathrm{w}}R_{2\mathrm{all}}$	0.0223	0.0274	0.0381	0.0420	0.0263	0.0311
GooF	1.039	1.037	1.054	1.036	1.046	1.010
CCDC #	2097610	2097611	2097612	2097613	2097614	2097615

**Table S1 (cont.). Crystallographic data and selected data collection parameters**			
**Cocrystal**	**(1-(2-oxopropyl)-1,4-dithianium)(triiodide) (7)**	**hydrogen-bis(1,4-dithiane-1,4-dioxide) tribromide (8)**	**(1,4-dithiane-1,4-dioxide) (hydronium)(bromide) (9)**
empirical formula	$C_7H_{13}I_3OS_2$	$C_4H_9Br_3O_2S_2$	$C_4H_{11}BrO_3S_2$
crystal system	monoclinic	triclinic	orthorhombic
space group	$P2_1/c$	*P*-1	*Pnma*
a (Å)	12.4057(6)	6.5374(3)	6.7267(2)
b (Å)	5.1987(3)	6.7536(3)	12.3074(4)
c (Å)	22.9962(11)	6.9275(3)	10.2186(3)
α (°)	90	61.560(2)	90
β (°)	101.190(2)	80.541(2)	90
γ (°)	90	75.375(2)	90
Volume (Å^3)	1454.91(13)	259.88(2)	845.98(5)
Z	4	1	4
D_c (g·cm^{-3})	2.548	2.511	1.972
μ (mm^{-1})	6.701	11.997	5.302
No. of ref. total	27970	7461	8435
No. of ref. unique	3458	1057	1041
No. of param.	119	65	56
R_1	0.0188	0.0117	0.0157
$R_{1\text{all}}$	0.0245	0.0294	0.0166
$_{\text{w}}R_2$	0.0375	0.0120	0.0413
$_{\text{w}}R_{2\text{all}}$	0.0436	0.0295	0.0420
GooF	1.112	1.072	1.050
CCDC #	2097616	2097617	2097618

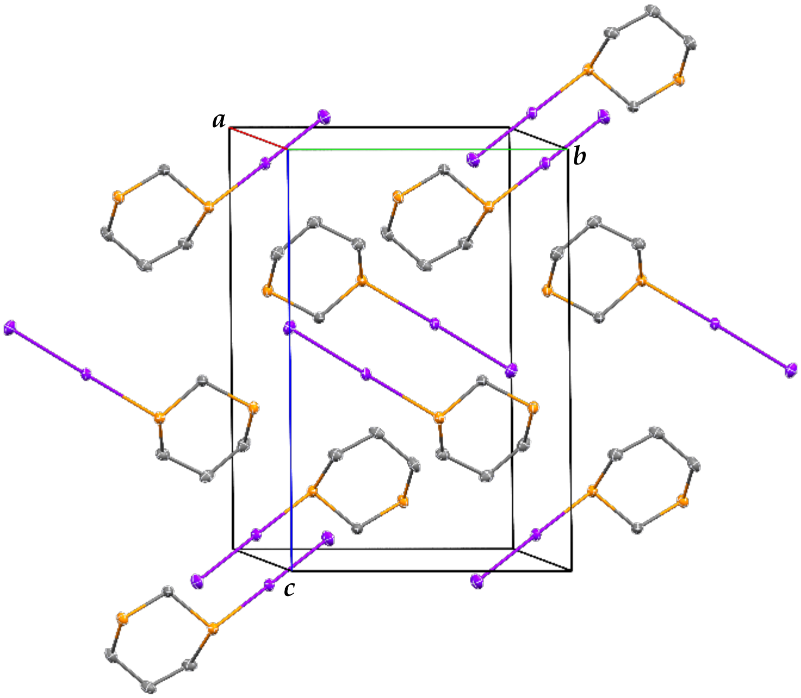

Figure S10. Unit cell packing diagram of **1**. Atomic displacement ellipsoids shown at the 50% probability level. Hydrogen atoms omitted for clarity.

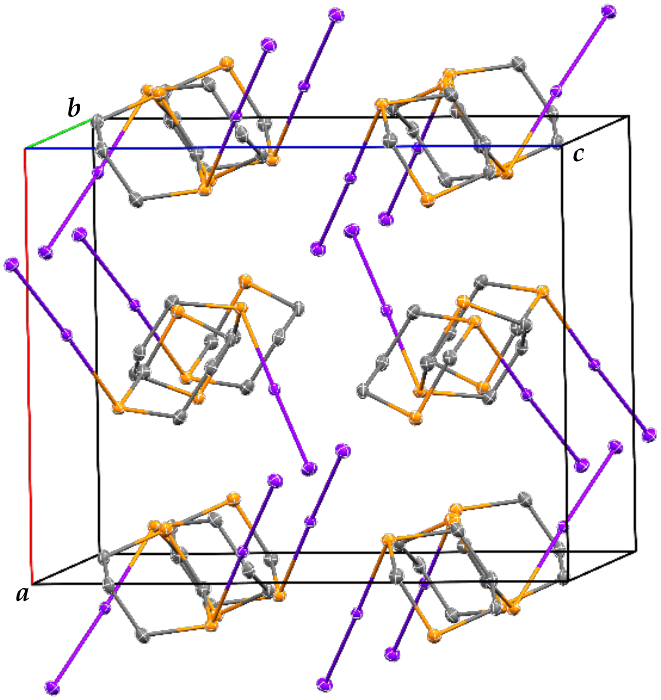

Figure S11. Unit cell packing diagram of **2**. Atomic displacement ellipsoids shown at the 50% probability level. Hydrogen atoms omitted for clarity.

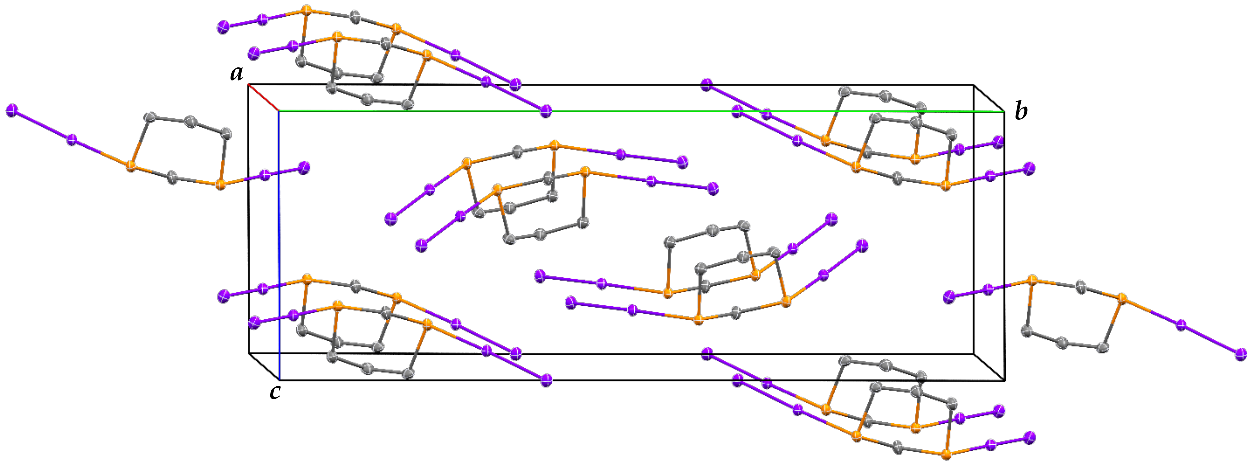

Figure S12. Unit cell packing diagram of **3**. Atomic displacement ellipsoids shown at the 50% probability level. Hydrogen atoms omitted for clarity.

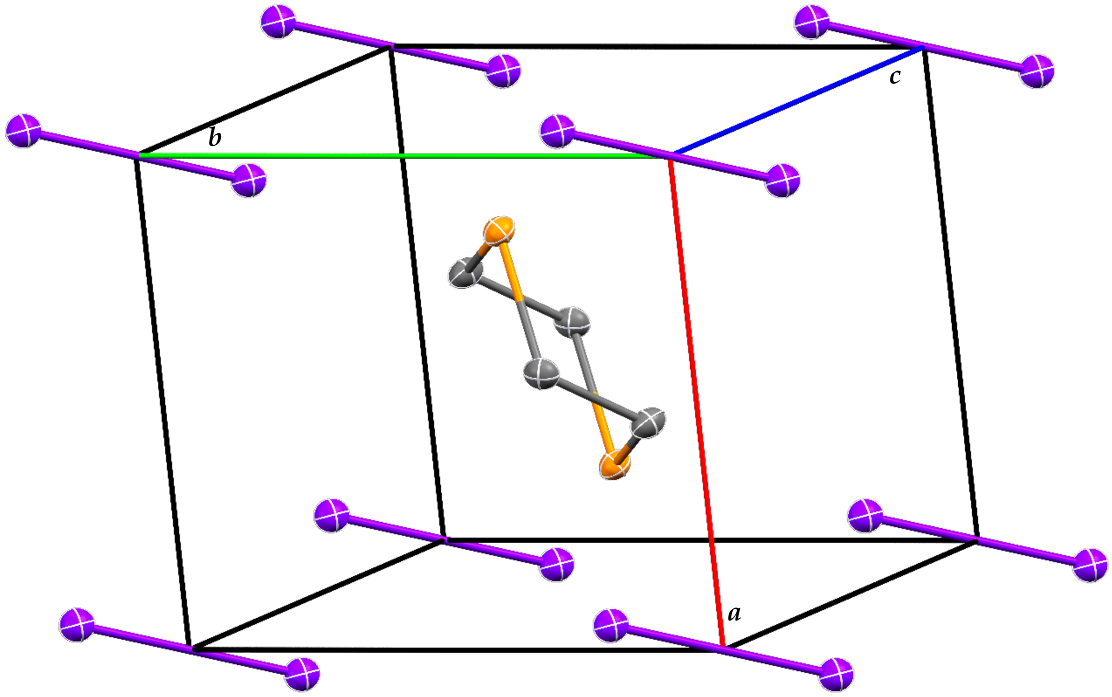

Figure S13. Unit cell packing diagram of **4**. Atomic displacement ellipsoids shown at the 50% probability level. Hydrogen atoms omitted for clarity.

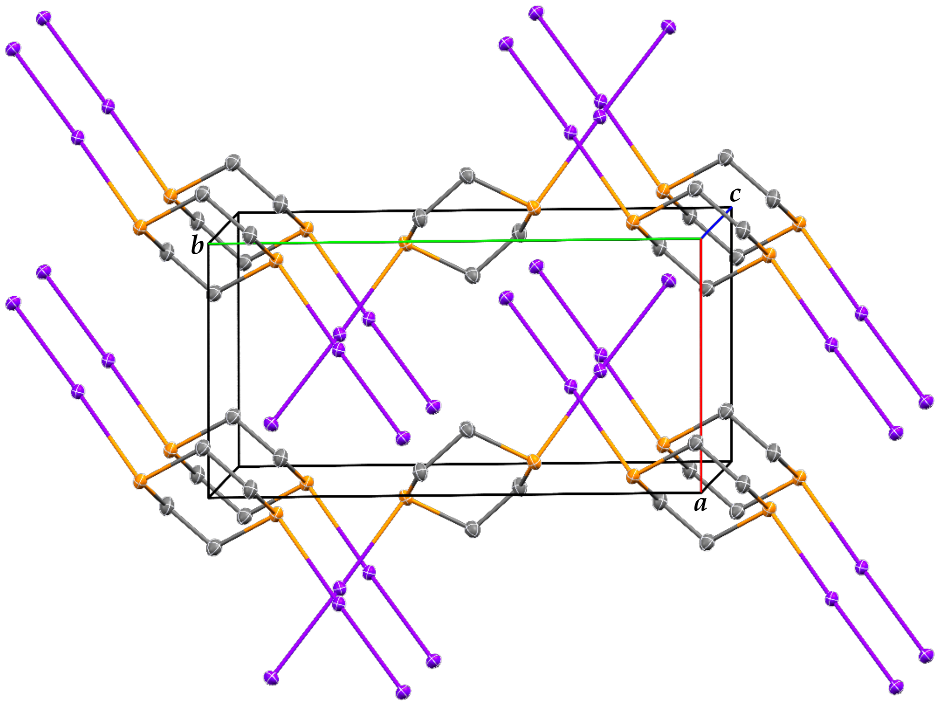

Figure S14. Unit cell packing diagram of **5**. Atomic displacement ellipsoids shown at the 50% probability level. Hydrogen atoms omitted for clarity.

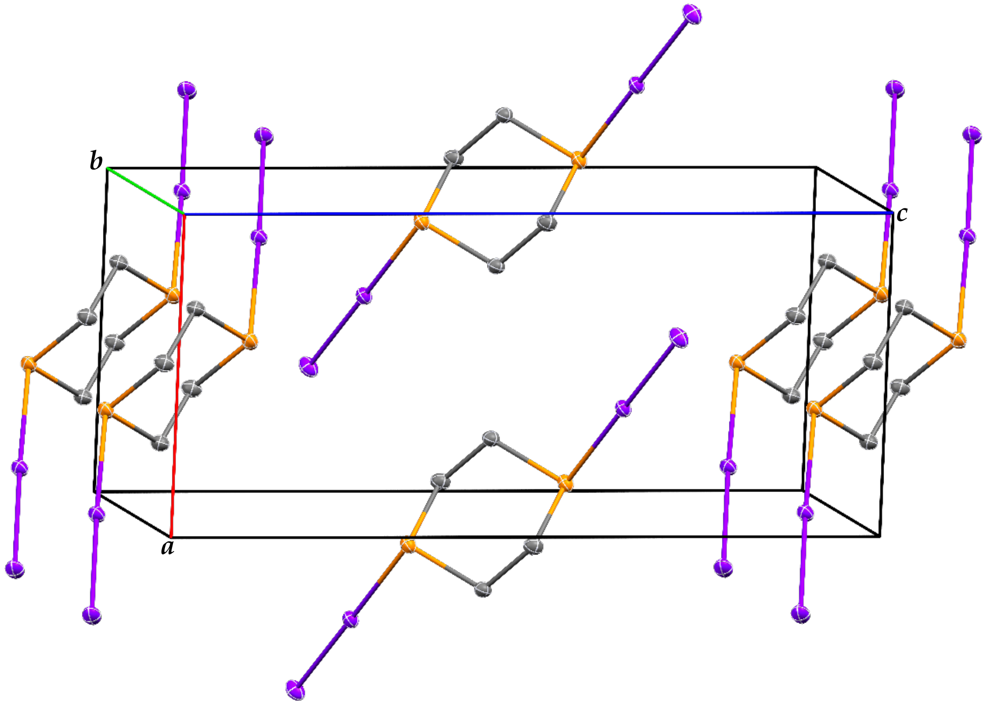

Figure S15. Unit cell packing diagram of **6**. Atomic displacement ellipsoids shown at the 50% probability level. Hydrogen atoms omitted for clarity.

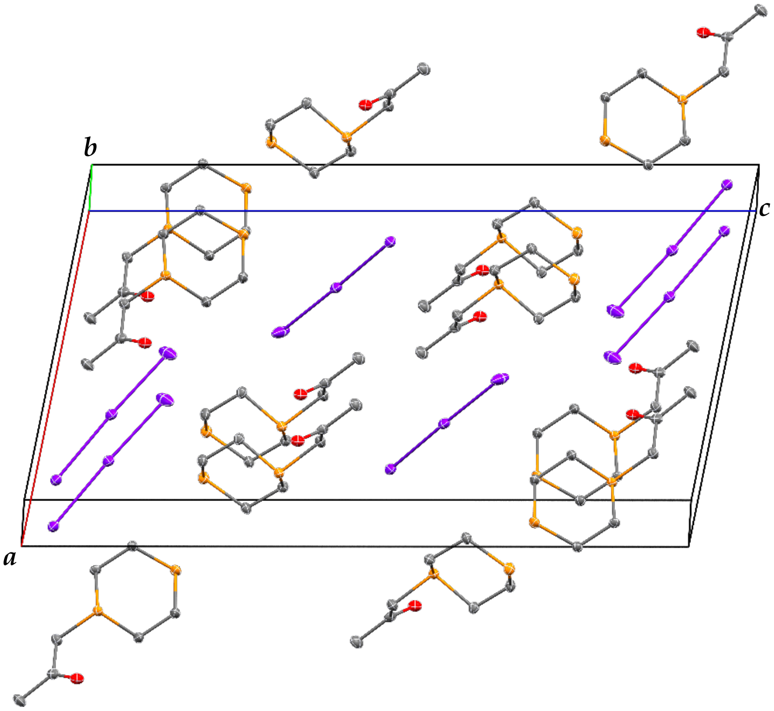

Figure S16. Unit cell packing diagram of **7**. Atomic displacement ellipsoids shown at the 50% probability level. Hydrogen atoms omitted for clarity.

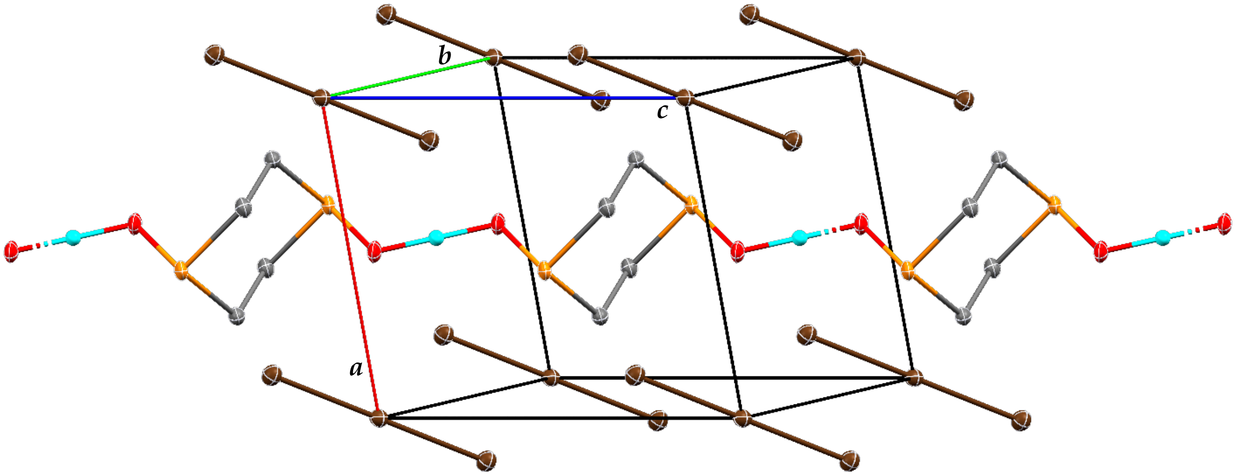

Figure S17. Unit cell packing diagram of **8**. Atomic displacement ellipsoids shown at the 50% probability level. Hydrogen atoms omitted for clarity, except those bound to oxygen atoms.

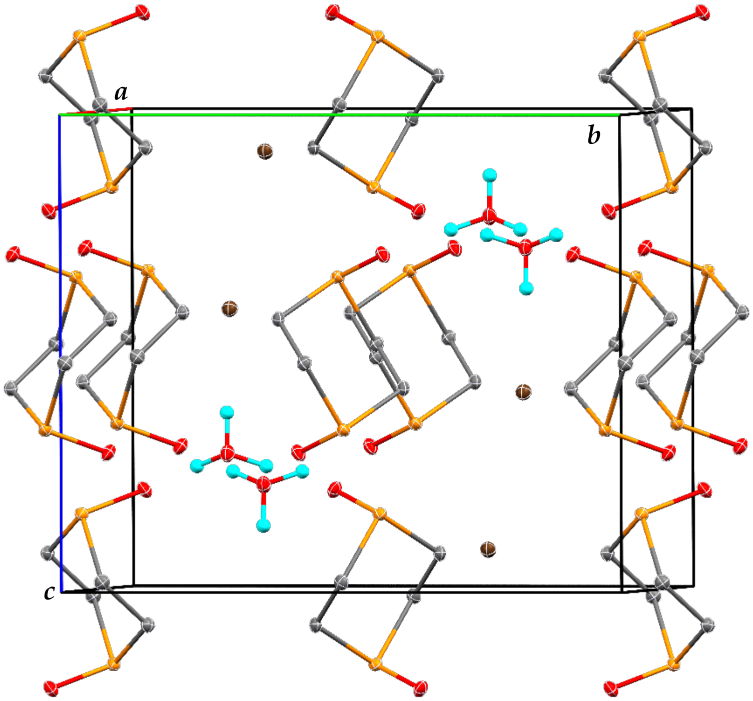

Figure S18. Unit cell packing diagram of **9**. Atomic displacement ellipsoids shown at the 50% probability level. Hydrogen atoms omitted for clarity, except those of the hydronium cations.
